# A chloroplast-targeted pentatricopeptide repeat protein PPR287 is crucial for chloroplast function and *Arabidopsis* development

**DOI:** 10.1186/s12870-019-1857-0

**Published:** 2019-06-07

**Authors:** Kwanuk Lee, Su Jung Park, Ji Hoon Han, Young Jeon, Hyun-Sook Pai, Hunseung Kang

**Affiliations:** 1 Department of Applied Biology, College of Agriculture and Life Sciences, Chonnam National University, 77 Yongbong-ro, Buk-gu, Gwangju, 61186 South Korea; 2 Department of Systems Biology, Yonsei University, Seoul, 03722 South Korea

**Keywords:** *Arabidopsis thaliana*, Chloroplast, Development, PPR, RNA metabolism

## Abstract

**Background:**

Even though the roles of pentatricopeptide repeat (PPR) proteins are essential in plant organelles, the function of many chloroplast-targeted PPR proteins remains unknown. Here, we characterized the function of a chloroplast-localized PPR protein (At3g59040), which is classified as the 287th PPR protein among the 450 PPR proteins in *Arabidopsis* (http://ppr.plantenergy.uwa.edu.au).

**Results:**

The homozygous *ppr287* mutant with the T-DNA inserted into the last exon displayed pale-green and yellowish phenotypes. The microRNA-mediated knockdown mutants were generated to further confirm the developmental defect phenotypes of *ppr287* mutants. All mutants had yellowish leaves, shorter roots and height, and less seed yield, indicating that PPR287 is crucial for normal *Arabidopsis* growth and development. The photosynthetic activity and chlorophyll content of *ppr287* mutants were markedly reduced, and the chloroplast structures of the mutants were abnormal. The levels of chloroplast rRNAs were decreased in *ppr287* mutants.

**Conclusions:**

These results suggest that PPR287 plays an essential role in chloroplast biogenesis and function, which is crucial for the normal growth and development of *Arabidopsis*.

**Electronic supplementary material:**

The online version of this article (10.1186/s12870-019-1857-0) contains supplementary material, which is available to authorized users.

## Background

The chloroplast genome encodes approximately 120–130 proteins necessary for photosynthesis and plastid biogenesis [1–3]. However, more than 3000 nucleus-encoded proteins are transported into the chloroplast, many of which are needed for chloroplast gene expression [4–7]. Chloroplast gene expression is modulated and regulated by posttranscriptional processes, such as mRNA and tRNA splicing, mRNA editing, RNA stability, and translational control, during which many RNA-binding proteins (RBPs) play essential roles [8–12]. In particular, most of the RBPs involved in chloroplast RNA metabolism are nucleus-encoded and are transported into chloroplasts [5–7, 13, 14].

The pentatricopeptide repeat (PPR) proteins are among the nucleus-encoded chloroplast RBPs. Notably, PPR proteins are particularly abundant in land plants [15–17]. The Arabidopsis genome encodes more than 450 PPR proteins, whereas less than 10 PPR proteins are found in humans [15, 18], suggesting their plant-specific functions. PPR proteins are divided into several subfamilies in terms of their tandem repeat motifs and additional domains. In general, the P-class PPR proteins that harbor only the PPR motifs are involved in intercistronic processing, splicing of group II introns, and RNA stabilization, whereas the PLS-class PPR proteins that contain additional C-terminal domains, such as E, E+, and DYW, are required for C to U RNA editing [17, 19, 20].

In chloroplasts of several plant species, including *Arabidopsis thaliana*, rice (*Oryza sativa*)*,* and maize (*Zea mays*)*,* the functions of many PPR proteins have been determined, and most of the chloroplast PPR proteins characterized so far participate mainly in the stabilization of mRNAs. Examples include CRP1 for stabilizing the 5′ and 3′ ends of the *petB*-*petD* intergenic region in maize [21, 22], PPR10 for stabilizing the 5′ and 3′ ends of the *atpI*-*atpH* and *psaJ*-*rpl33* intergenic regions in maize [23–25], HCF152 for stabilizing the 5′ and 3′ ends of the *psbH*-*petB* intergenic region in *Arabidopsis thaliana* [26, 27], MRL1 for stabilizing the *rbcL* 5′ end in *Arabidopsis* [28], and PGR3 for stabilizing the *petL* 5′ end in *Arabidopsis* [29, 30]. Similarly, mitochondrial PPR19 and MTSF1 are needed for stabilizing the *nad1* intron 3′ end and *nad4* 3′ end in *Arabidopsis* [31, 32]. Analysis of loss-of-function mutants demonstrated that these aforementioned PPR proteins are critical for normal growth and development of plants. Although these previous studies clearly show that PPR proteins are essential for organellar functions and plant development, the cellular functions of many PPR proteins still remain to be characterized.

In this study, we determined the function of a chloroplast-targeted PPR protein possessing 10 PPR motifs (At3g59040), which is classified as the 287th PPR protein among the 450 PPR proteins in *Arabidopsis* (http://ppr.plantenergy.uwa.edu.au) [33], thus designated PPR287. We show that PPR287 affects the level of chloroplast rRNAs, which is essential for chloroplast biogenesis and function as well as for the normal growth and development of *Arabidopsis*.

## Results

### Cellular localization and expression patterns of PPR287

**Fig. 1:**
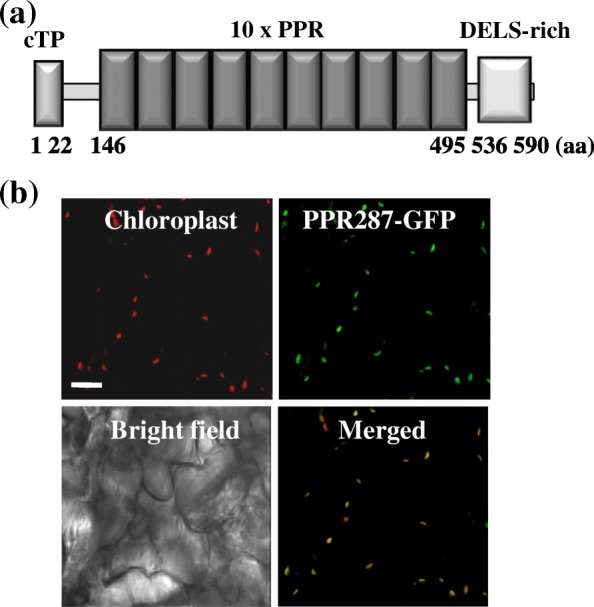
The domain structure and cellular localization of PPR287. **a** Schematic representation of the PPR287 domain structures. The chloroplast transit peptide (cTP) and PPR motifs are shown. The number of amino acid (aa) in PPR287 is indicated, and a region rich in aspartate (D), glutamate (E), leucine (L), and serine (S) residues at the C-terminus is indicated. **b** Cellular localization of the PPR287 protein. GFP signals from the PPR287-GFP-expressing transgenic Arabidopsis were observed using a confocal microscope. Red signals indicate chloroplast auto-fluorescence. Bar = 10 μm

PPR287 harbors a putative chloroplast transit peptide at the N-terminus, ten tandemly repeated PPR motifs comprising from 146 to 495 amino acids, and a region rich in aspartate, glutamate, leucine, and serine residues at the C-terminus (Fig. 1a). To verify whether PPR287 is indeed transported into chloroplasts, transgenic *Arabidopsis* plants that express the PPR287-green fluorescent protein (GFP) fusion protein or only the GFP protein as a control were generated and analyzed by means of confocal microscopy. Evidently, GFP signals were observed in the chloroplasts of PPR287-GFP expressing transgenic plants (Fig. 1b), whereas GFP signals were detected in the nucleus and cytoplasm of GFP-expressing transgenic plants (Additional file 1), indicating that the nucleus-encoded PPR287 is transported into chloroplasts. To identify the tissue-specific expression patterns of *PPR287*, an approximately 1.5-kb fragment of the genomic DNA harboring the putative promoter of PPR287 was cloned in front of a GUS reporter gene, and the PPR287_PRO_::GUS expression was observed in transgenic *Arabidopsis* plants. Strong GUS signals were detected in young seedlings, leaves, and flowers, whereas only weak GUS signals were observed in stems and siliques (Additional file 2).

### PPR287 is essential for normal growth and development of *Arabidopsis*

**Fig. 2:**
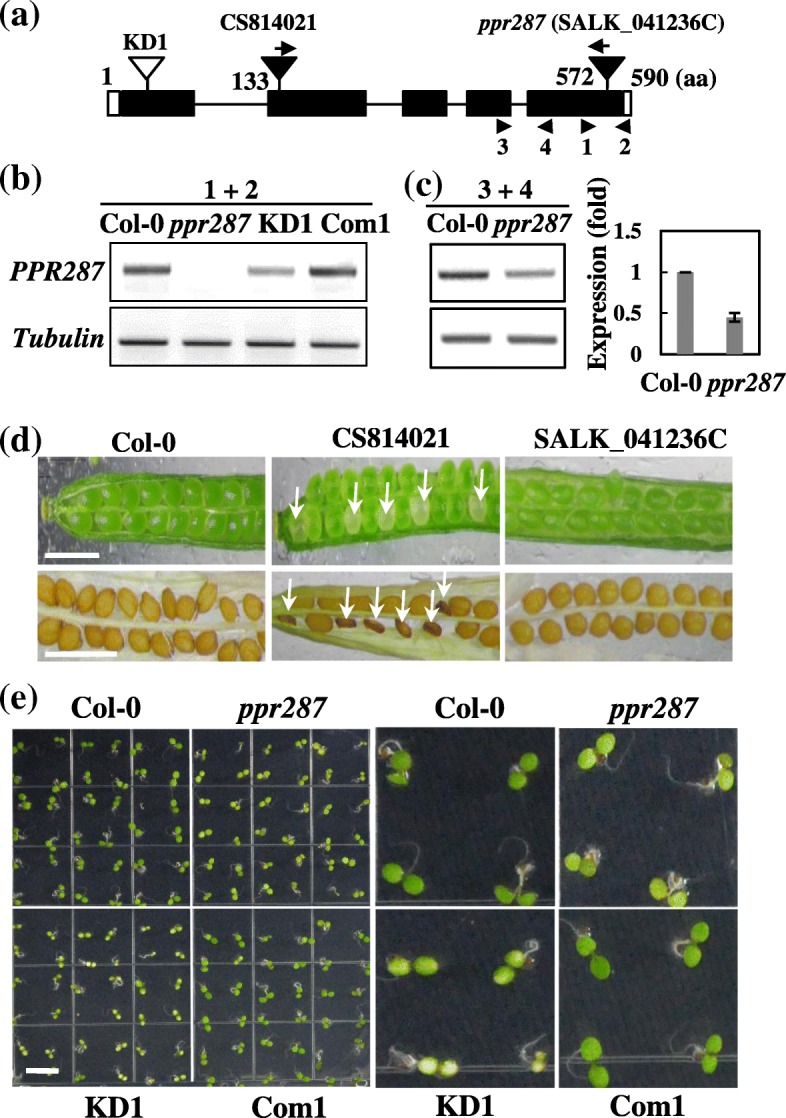
Development-defect phenotypes of the *ppr287* mutants. **a** Schematic representation of T-DNA insertion sites and amiRNA knockdown mutant (KD1) target site. Black rectangles and lines represent exons and introns, respectively, and untranslated regions are indicated by white rectangles. The positions of T-DNA insertion in the *ppr287* mutants and amiR target site are indicated by black and white triangles, respectively. The number of amino acid (aa) in PPR287 is indicated. Positions of primers used for RT-PCR in (**b** and **c**) are shown with arrowheads. **b** RT-PCR analysis confirming the absence or downregulation of *PPR287* expression in the *ppr287* mutant, amiR mutant (KD1), and complementation line (Com1) in *ppr287* mutant background. The primer pair (1 + 2) amplifying the *PPR287* transcript spanning the T-DNA insertion site was used. **c** RT-PCR (left) and quantitative real-time RT-PCR (right) analyses confirming the downregulation of *PPR287* expression in the *ppr287* mutant. The primer pair (3 + 4) amplifying the *PPR287* transcript upstream of the T-DNA insertion site was used. Tubulin was used as a loading control. **d** Seed development of the heterozygous mutant. White arrows indicate aborted seeds in the mutants. Bar = 1 cm. **e** Pale-green phenotypes of the *ppr287* and KD1 line observed at 6 days after germination

To identify the function of PPR287 in plant growth and development, two T-DNA insertion *Arabidopsis* mutant lines were obtained and analyzed. The CS814021 line has T-DNA inserted into the second exon (Fig. 2a); about a fourth of its seeds were aborted and shriveled (Fig. 2d). Importantly, the homozygous mutant of the CS814021 line could not be obtained.

All CS814021 mutant seeds that were germinated on MS medium containing sucrose were heterozygotes. Many CS814021 mutant seeds were not germinated on sucrose-containing MS medium, which are supposed to be homozygotes. These results suggest that PPR287 is essential for embryogenesis. In contrast, a homozygous mutant of the SALK_041236C line, in which T-DNA was inserted at the position encoding 572th amino acid near the stop codon in the last exon of PPR287 (Fig. 2a), could be obtained. No band corresponding to *PPR287* was detected in the *ppr287* mutant by RT-PCR using the primer pair amplifying the *PPR287* transcript spanning the T-DNA insertion site (Fig. 2b), suggesting knockout mutant. However, because the T-DNA is inserted downstream of the PPR motif (Fig. 2a), it is possible that partially truncated PPR287 protein containing entire PPR motifs can be expressed in the *ppr287* mutant, suggesting the *ppr287* mutant is not a genuine loss-of-function mutant. To examine whether T-DNA insertion affects the transcript level of *PPR287* in the mutant, the level of *PPR287* in the mutant was analyzed by RT-PCR and quantitative real-time RT-PCR using the primer pair amplifying the *PPR287* transcript upstream of the T-DNA insertion site (Fig. 2a). Cleary, the *PPR287* level was decreased down to approximately 50% of the wild-type level (Fig. 2c), confirming that the *ppr287* is a knockdown mutant.

**Fig. 3:**
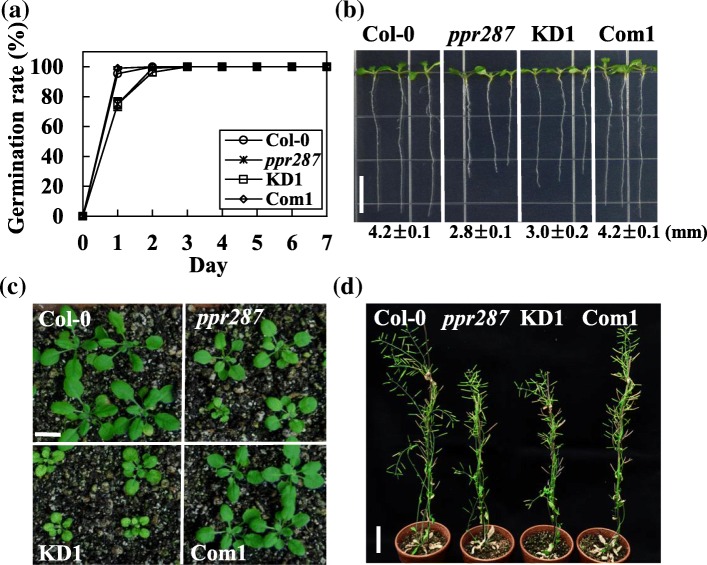
Growth-defective phenotypes of the *ppr287* mutant. **a** Seed germination rates of the Col-0, *ppr287* mutant, amiRNA knockdown mutant (KD1), and complementation line (Com1) were analyzed on the indicated days. **b** Root lengths of the plants were measured 10 days after germination (DAG). Data are mean ± SD of three biological replicates (*n* = 5). Bar = 1 cm. **c** Growth of the plants at 20 DAG. Bar = 1 cm. **d** Height of the plants at 50 DAG. Bar = 10 cm

Because the loss-of-function of *PPR287* leads embryo lethality, we generated *ppr287* knockdown mutants by means of an artificial microRNA (amiRNA)-mediated knockdown method. The transgenic *Arabidopsis* plants expressing the amiRNA, which was designed to cleave the first exon of *PPR287*, were generated, and downregulation of *PPR287* in each transgenic line was confirmed by RT-PCR and real-time RT-PCR analysis (Fig. 2b and Additional file 3). The amiRNA knockdown (KD) lines as well as the *ppr287* mutant displayed development-defect phenotypes, such as yellowish and pale-green leaves (Fig. 2e). Interestingly, the severity of developmental defects was closely correlated to the knockdown levels of *PPR287*; the KD1 line, in which the *PPR287* level was decreased down to approximately 30% of the wild-type level, exhibited the most severe abnormal development, whereas the KD2 and KD3 lines, in which *PPR287* levels were approximately 40 to 60% of the wild-type levels, showed mild abnormal development (Additional file 3). Among the three knockdown lines, the KD1 line showing obvious phenotypes was chosen for further analysis. To further confirm the function of PPR287, a complementation line expressing PPR287 in the *ppr287* mutant background (Com1/*ppr287*) was also generated and analyzed. The expression of *PPR287* in the complementation line (Com1) was confirmed by RT-PCR (Fig. 2b). Abnormal development was observed in both the knockout *ppr287* and the knockdown KD1 mutants, whereas the complementation line exhibited wild-type phenotypes (Fig. 2e). Germination of the mutant seeds was slightly retarded (Fig. 3a), and root growth of the mutants was also more inhibited than that of the wild type (Fig. 3b). The delayed growth and development of the mutants were much more evident when grown in soil (Fig. 3c and d). The plantlets and leaves of the mutants were much smaller than those of the wild type (Additional file 4). Bolting time was delayed in the mutants, but the number of leaves at bolting was not different between the wild-type and mutant plants (Additional file 4), suggesting that PPR287 does not affect flowering time. Plant height and seed yield were significantly decreased in the mutants compared with the wild type (Additional file 4). Collectively, these results suggest that PPR287 is essential for normal growth and development of *Arabidopsis*.

### PPR287 affects photosynthetic activity and chloroplast biogenesis

**Fig. 4:**
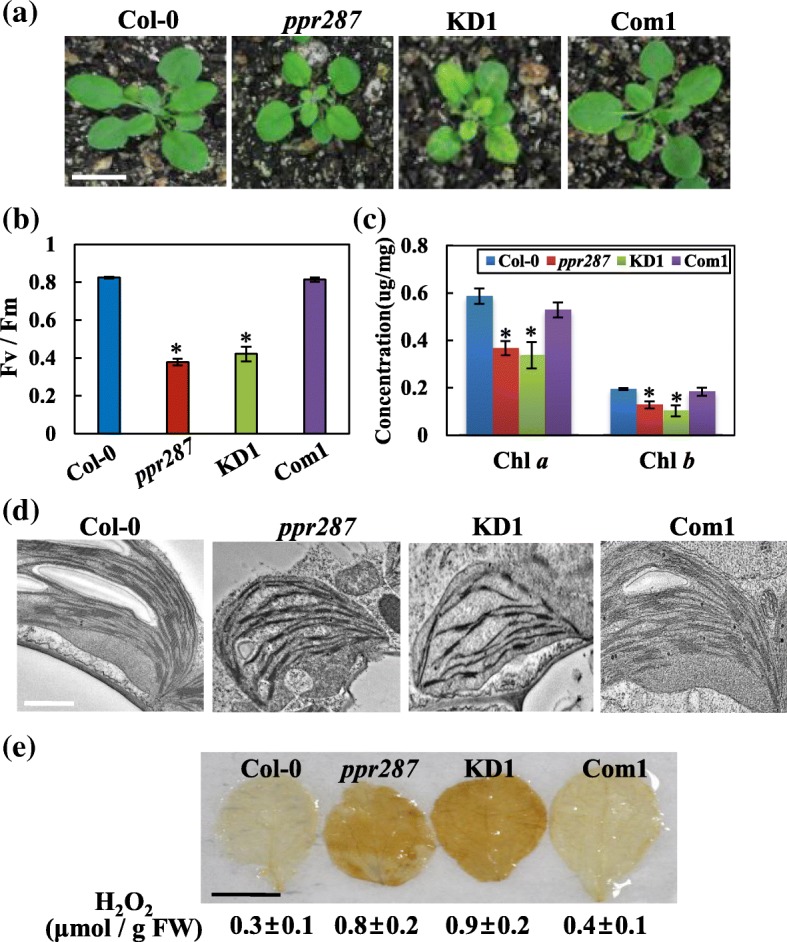
PPR287 is crucial for photosynthesis and chloroplast biogenesis. **a** Pale-green phenotypes of the *ppr287* mutant and amiRNA knockdown mutant (KD1) observed at 20 days after germination (DAG). Bar = 1 cm. **b** Chlorophyll fluorescence (Fv/Fm) and (**c**) chlorophyll contents in each plant at 10 DAG. Data are mean ± SD of three biological replicates (n = 5), and statistically different values are indicated by asterisks (*p* ≤ 0.05). **d** Chloroplast structures in the leaves of 3-week-old plants were observed using a transmission electron microscope. Bar = 1 μm. **e** In situ detection of H_2_O_2_ levels in the leaves of each plant were performed by DAB (3,3′-diaminobenzidine) staining. Bar = 5 mm. The levels of H_2_O_2_ were quantified, and data are mean ± SD of three biological replicates (n = 5). FW, fresh weight

Because *ppr287* mutants showed retarded growth and pale-green phenotypes (Fig. 4a), we investigated whether PPR287 affects photosynthetic activity, chlorophyll content, and chloroplast biogenesis. It was evident that photosynthetic activity, as shown by maximum quantum yield of photosystem II (Fv/Fm), was significantly reduced in *ppr287* mutants compared with the wild type (Fig. 4b). Chlorophyll contents in the mutants were much less than those in the wild type (Fig. 4c). Photosynthetic activity and chlorophyll content of the complementation line recovered to wild-type levels. These results suggest that PPR287 plays an essential role in photosynthetic activity and chlorophyll biosynthesis.

We next examined whether PPR287 is involved in chloroplast biogenesis by observing chloroplast structures using a transmission electron microscope. Chloroplast morphology was abnormal in *ppr287* mutants, displaying a few and loosely stacked thylakoids, whereas the wild type and the complementation line had normal chloroplasts (Fig. 4d and Additional file 5). To identify whether this abnormal chloroplast structure affects chloroplast function, we analyzed the levels of reactive oxygen species (ROS), which are an indicator of chloroplast function. Among ROS, such as superoxide (O_2_^−^), hydrogen peroxide (H_2_O_2_), and nitric oxide (NO), the amount of H_2_O_2_ was measured using a 3,3′-diaminobenzidine (DAB) staining. Evidently, *ppr287* mutants had much stronger DAB staining than the wild type and complementation line (Fig. 4e). These results were further supported by quantifying H_2_O_2_ levels in the leaves of each plant. The levels of H_2_O_2_ were much higher in *ppr287* mutants than the wild type and complementation line (Fig. 4e). Collectively, these results imply that PPR287 is important for photosynthesis and chloroplast biogenesis and function.

### PPR287 affects transcript levels of chloroplast rRNAs

**Fig. 5:**
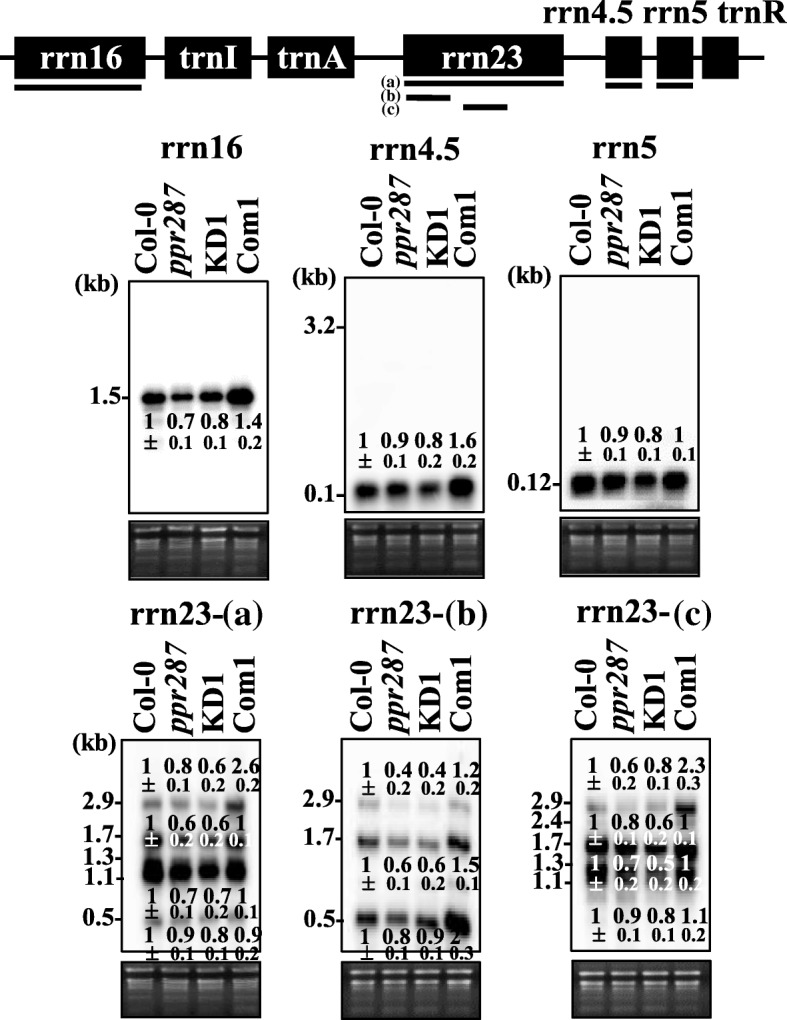
Decreased levels of chloroplast rRNAs in the ppr287 mutant. Total RNA extracted from 1-week-old Col-0, ppr287 mutant, amiRNA knockdown mutant (KD1), and complementation line (Com1) was separated on a 1.2% formaldehyde agarose gel, and the levels of the processed products were determined by Northern blot analysis using the probes represented by thick lines below each gene; three probes for rrn23 are labeled with (**a**), (**b**), and (**c**). Relative intensities of rRNA bands in each sample were calculated, and the values under each lane are mean ± SD of three replicates

The next important task is to determine how PPR287 affects chloroplast biogenesis and function. Because diverse PPR proteins are involved in organellar RNA metabolism, including intron splicing and RNA stability, we first analyzed whether PPR287 is involved in the splicing of chloroplast introns. The results showed that the splicing efficiencies of none of the intron-containing chloroplast mRNAs and tRNAs were altered in *ppr287* mutants (Additional file 6), suggesting that PPR287 is not involved in the splicing of chloroplast introns. Next, we analyzed whether PPR287 affects the expression of chloroplast genes. The levels of none of the chloroplast transcripts were altered in *ppr287* mutants (Additional file 7), suggesting that PPR287 does not affect the expression of chloroplast genes. Finally, we investigated whether PPR287 is involved in chloroplast rRNA stability by northern blot analysis. The precursor and mature products of 4.5S, 5S, 16S, and 23S rRNAs were detected with the probes specific to each rRNA. The results showed that the levels of all rRNAs were decreased in *ppr287* mutants. Notably, both the precursor and mature products of 23S rRNA were decreased in the mutants (Fig. 5). The decreased intensities of rRNA products observed in *ppr287* mutants recovered to normal level in the complementation line. These results suggest that PPR287 may affect rRNAs stability in chloroplasts.

## Discussion

Contrary to the increasing understanding of the distribution and organellar targeting of PPR proteins in higher plants, the function and cellular role of only a few PPR proteins have been determined until recently. Our current results demonstrate that PPR287 plays a crucial role in *Arabidopsis* development by affecting the level of chloroplast rRNAs. No loss-of-function homozygous mutants of Arabidopsis PPR287 could be obtained (Fig. 2), suggesting that PPR287 is essential for plant development. In contrast, the homozygous mutant of PPR287 that has T-DNA inserted near the stop codon in the last exon of PPR287 (Fig. 2) could be obtained, which showed pale-green phenotypes but developed normal seeds (Fig. 2). This mutant presumably behaves like a knockdown mutant because it can synthesize partially truncated functional proteins (Fig. 2). This hypothesis was further supported by the observation that *ppr287* knockdown mutants generated by an amiRNA knockdown method exhibited similar pale-green phenotypes. Both knockdown mutants displayed severe defects in leaf greening, photosynthesis, chlorophyll biosynthesis, and chloroplast biogenesis (Figs. 2, 3 and 4).

Notably, PPR287 affects transcript levels of chloroplast rRNAs, which then affects chloroplast biogenesis and function. When the processing of rRNAs is impaired, the mature rRNAs are decreased, with concomitant increase in the precursor rRNAs, as exemplified in other PPR mutants, such as *svr7* and *sot1* [34, 35]. Because our northern blotting analysis revealed that the intensities of precursor rRNAs were not increased in *ppr287* mutants, but both the precursor and mature products of all chloroplast rRNAs were decreased in the mutants (Fig. 5a), we propose that PPR287 affects the stability of chloroplast rRNAs. Although we cannot rule out the possibility that the lower amount of rRNAs in *ppr287* mutants is due to the lower transcription of rRNA genes, it is unlikely that PPR287, as an RNA-binding protein, affects transcription of rRNA genes. Contrary to many reports demonstrating the roles of PPR proteins in the stabilization of chloroplast mRNAs, the function of PPR proteins involved in the stabilization of rRNAs is largely unknown. A previous study has shown that maize PPR53, which is orthologous to the Arabidopsis PPR protein SOT1, enhances the stability of chloroplast 23S rRNA [36]. Our current results add PPR287 as another PPR protein possibly involved in the stabilization of chloroplast rRNAs. However, we do not know at present the mechanistic role of PPR287 affecting the level of chloroplast rRNAs. Given that plastid rRNA operon encodes all plastid rRNAs and two tRNAs (trnI and trnA) as a single transcript unit (Fig. 5), which is then processed to each mature transcript, it is likely that PPR287 binds to the primary or precursor rRNA transcript and thereby stabilizes all chloroplast rRNAs (Fig. 5). More analysis is required to identify the binding sites of PPR287 and the effects of PPR287 binding on the stabilization of chloroplast rRNAs. In particular, it would be necessary to determine whether PPR287 binds to the specific sequence in the 5′ end or 3′ end of plastid primary rRNA transcript and thereby affects the stability of all rRNAs or whether PPR287 stabilizes all chloroplast rRNAs by binding to the conserved sequences in each rRNA transcript. In addition, it would be interesting to investigate whether PPR287 interacts with other proteins, which affects the level and stability of chloroplast rRNAs. These key experiments will greatly contribute to fully understand the cellular role of PPR287 in the stabilization of chloroplast rRNAs.

## Conclusions

Our results demonstrate that the chloroplast-transported PPR287 affects transcript levels of chloroplast rRNAs, which is crucial for chloroplast biogenesis and function during plant growth and development. Given that the role of PPR proteins in the stabilization and processing of chloroplast rRNAs has been identified in only a few cases, our results demonstrating that PPR287 affects the level of all chloroplast rRNAs are intriguing. Characterizing the effect of PPR287 binding on the stabilization of chloroplast rRNAs should be a next important experiment. Moreover, further research is needed to identify the functions of many as-yet uncharacterized PPR proteins and their coordinated roles in the splicing, processing, and stability of chloroplast transcripts.

## Methods

### Plant materials, mutants, and growth conditions

The wild-type and mutant *A. thaliana* were Col-0 ecotype. The *Arabidopsis* T-DNA insertion mutants, CS814021 and SALK_ 041236C, were obtained from the Arabidopsis Biological Resources Center. The *ppr287* knockdown mutants were generated using an artificial microRNA-mediated knockdown method [37, 38]. The amiRNA constructs targeting the first exon of *PPR287* were designed using the Web MicroRNA Designer program (http://wmd3.weigelworld.org) and were cloned into the pBI121 vector. *Arabidopsis* transformation was performed by means of vacuum infiltration using *Agrobacteruim tumefaciens* GV3101 [39]. Complementation lines were generated by expressing the full-length PPR287 under the control of the cauliflower mosaic virus 35S promoter in the *ppr287* mutant background. The T_3_ or T_4_ homozygote transgenic lines were selected for phenotypic analysis. The expression levels of *PPR287* in knockdown mutants and complementation lines were analyzed by RT-PCR with gene-specific primers listed in Additional file 8. All plants were grown in soil or half-strength Murashige and Skoog (MS) medium containing 1% sucrose at 23 ± 2 °C under long-day conditions (16 h-light / 8 h-dark cycle).

### Analysis of subcellular localization of PPR287

The cDNA encoding full-length PPR287 was cloned into the *Xba*I/*Eco*RI site of CsV-GFP3-PA vector using the primers listed in Additional file 8, which expresses the PPR287-GFP fusion protein under the control of cassava vein mosaic virus promoter. Transgenic *Arabidopsis* plants that express the PPR287-GFP fusion protein were generated by means of vacuum infiltration using *Agrobacteruim tumefaciens* GV3101 [39], and GFP signals were detected using a Zeiss LSM510 laser scanning confocal microscope (Carl Zeiss Inc. Thornwood, NY, USA). The excitation and emission wavelengths were 488 and 505 nm, respectively.

### Chlorophyll content and chlorophyll fluorescence measurement

Chlorophyll content was measured using ethanol extraction and quantification method as previously described [40]. Briefly, leaves of one-week-old wild-type, mutants, and complementation lines were ground in liquid nitrogen, and chlorophyll was extracted with 96% ethanol. The samples were kept overnight at room temperature in the dark. After centrifugation, the absorbance of the supernatant was measured at 648 nm and 664 nm. Chlorophyll fluorescence (Fv/Fm) was measured with a Handy PEA chlorophyll fluorimeter according to the manufacturer’s instructions (Hansatech Instruments Ltd., Norfolk, UK).

### Transmission electron microscopy

Chloroplast structures were analyzed using TEM as previously described [41, 42]. Briefly, two-week-old seedlings were fixed with a mixture of 2% glutaraldehyde and 2% paraformaldehyde in 50 mM cacodylate buffer, pH 7.2, at room temperature for 4 h. The samples were embedded in LR White (London Resin Co., London, UK) at 50 °C for 24 h, and thin sections (80–100 nm thickness) were prepared using an ultra-microtome with a diamond knife. The thin sections were stained with uranyl acetate and lead citrate and then examined using a transmission electron microscope JEM-1400 (Jeol, Tokyo, Japan).

### RNA extraction, RT-PCR, and northern blot analysis

Total RNA was isolated from the frozen samples using the Plant RNeasy extraction kit (Qiagen, Valencia, CA, USA). The splicing pattern of the intron-containing genes was analyzed by RT-PCR using the gene-specific primers listed in Additional file 8 as previously described [43]. Splicing efficiency was measured by real-time RT-PCR using the gene-specific primers listed in Additional file 9 as previously described [32, 41]. The levels of chloroplast transcripts were measured by quantitative RT-PCR using the gene-specific primers listed in Additional file 10. Real-time RT-PCR was carried out on a Rotor-Gene Q thermal cycler (Qiagen) using a SYBR Green RT-PCR kit (Qiagen). For northern blot analysis, four or five micrograms of total RNA were separated on a 1.2% formaldehyde-agarose gel and transferred to a Hybond-N^+^ nylon membrane (Amersham Biosciences, Parsippany, NJ, USA). The [α-^32^P]-labeled probes were synthesized using a random primer DNA labeling kit (TaKaRa Bio., Shiga, Japan). Hybridization, washing, and detection of signals were performed essentially as described previously [32].

### GUS staining, DAB staining, and H_2_O_2_ measurement

To examine the tissue-specific expression patterns of *PPR287*, an approximately 1.5-kb fragment of the genomic DNA harboring the putative promoter of PPR287 was cloned into the *Sph*I/*Bam*HI site in front of a GUS reporter gene in pBI121 vector using the primers listed in Additional file 8, and the resulting PPR287_PRO_::GUS construct was introduced into *Arabidopsis* by means of vacuum infiltration using *Agrobacteruim tumefaciens* GV3101 [39]. The transgenic plants were stained in a 0.5 M potassium phosphate buffer (pH 7.0) solution containing 0.5 mM potassium ferrocyanide, 0.5 mM potassium ferricyanide, 10 mM EDTA, 1 mg/ml 5-bromo-4-chloro-3-indole-β-D-glucuronide, 20% methanol, and 0.05% Triton X-100 at 37 °C for 24 h in the dark. After washing the samples with ethanol, GUS images were observed using a Zeiss Axioplan microscope (Carl Zeiss, Inc.). In situ detection of H_2_O_2_ was carried out by 3,3′-diaminobenzidine (DAB) staining as previously described [44]. Briefly, 2-week-old leaves were immersed in a solution (0.1% DAB, 0.01 M Na_2_HPO_4_, pH 3.8, 0.05% Tween-20), vacuum-infiltrated for 10 min, and incubated overnight at room temperature in the dark. Photographs were taken after bleaching out chlorophylls in an 80% ethanol solution. The level of H_2_O_2_ was measured as previously described [45]. Briefly, approximately 200 mg of tissue samples were treated with 0.1% trichloroacetic acid at 4 °C, and the extract was mixed with 100 mM potassium phosphate buffer (pH 7.0) and 1 M KI solution. The reaction mixture was placed in the dark at room temperature for 1 h, and the H_2_O_2_ content was determined by measuring the absorbance at 410 nm.

### Statistical analysis

The differences in growth parameters, chlorophyll contents, and photosynthetic activity between the wild type, mutants, and complementation lines were compared by *t* test (*p* ≤ 0.05) using the SigmaPlot 10 program (Systat Software, Inc., San Jose, CA, USA).

## Additional files

Additional file 1: Cellular localization of the GFP only protein. GFP signals from the GFP-expressing transgenic Arabidopsis plants were observed using a confocal microscope. Red signals indicate chloroplast auto-fluorescence. Bar = 10 μm. (PDF 52 kb)

Additional file 2: Tissue-specific expression patterns of *PPR287*. GUS activity in (a) 5-day-old seedling, (b) 14-day-old seedling, (c) 30-day-old leaf, (d) 40-day-old stem, (e) and (f) flowers, (g) stigma, and (h) siliques. Bar = 1 cm. (PDF 49 kb)

Additional file 3: Development-defect phenotypes of artificial microRNA-mediated PPR287 knockdown mutants. (a) Schematic representation of the amiRNA and its target sequence. (b) Downregulation of *PPR287* in each knockdown mutant line was determined by RT-PCR and real-time PCR. (c) Phenotypes of knockdown mutants on MS media at 7 days. Bar = 1 cm. (PDF 120 kb)

Additional file 4: PPR287 plays a role in plant growth and development. (a) Bolting time, (b) leaf number at bolting, (c) plant height, and (d) seed yield of the Col-0, *ppr287* mutant, knockdown mutant (KD1), and complementation line (Com1). Values are mean ± SE of three independent experiments (*n* = 5), and statistically different values are indicated by asterisks (*P* ≤ 0.05). (e) Growth and leaf morphology at 27 days. Bar = 1 cm. (PDF 161 kb)

Additional file 5: Abnormal chloroplast structures in *ppr287* mutants. The Col-0, *ppr287* mutant, knockdown mutant (KD1), and complementation line (Com1) were grown on MS medium, and chloroplast structures in the leaves of 3-week-old plants were observed using a transmission electron microscope. Bar = 1 μm. (PDF 177 kb)

Additional file 6: Splicing patterns of intron-containing chloroplast genes. (a) Total RNA was extracted from 2-week-old Col-0 (W), *ppr287* mutant (K), knockdown mutant (KD1), and complementation line (Com1), and transcript levels of each gene were analyzed by RT-PCR. (b) The rations of un-spliced (precursor) to spliced (mature) transcripts between mutant and wild-type were determined by real-time RT-PCR. Data are mean ± SE of three independent biological replicates. (PDF 81 kb)

Additional file 7: Expression levels of chloroplast genes. Total RNA was extracted from 2-week-old Col-0, *ppr287* mutant, and knockdown mutant (KD1), and transcript levels of chloroplast genes were analyzed by real-time RT-PCR. Data are mean ± SE of three independent biological replicates (PDF 28 kb)

Additional file 8: Gene-specific primer used in RT-PCR experiments and vector construction (PDF 114 kb)

Additional file 9: Gene-specific primers used for the analysis of splicing efficiency by real-time RT-PCR. (PDF 143 kb)

Additional file 10: Gene-specific primers used for the analysis of expression levels of chloroplast genes by quantitative RT-PCR. (PDF 234 kb)

## Data Availability

All data can be found within the manuscript and additional files. The datasets used and/or analyzed during the current study are available from the corresponding author on reasonable request.

## Abbreviations

amiRNA: artificial microRNA
Com: Complementation line
DAB: 3,3′-diaminobenzidine
Fv/Fm: quantum yield of photosystem II
GFP: Green fluorescent protein
H_2_O_2_: Hydrogen peroxide
KD: Knockdown
MS: Murashige and Skoog
NO: Nitric oxide
O_2_^−^: superoxide
PPR: Pentatricopeptide repeat
RBP: RNA-binding protein
ROS: Reactive oxygen species
