# Supplementary figures and images for: A chloroplast-targeted pentatricopeptide repeat protein PPR287 is crucial for chloroplast function and Arabidopsis development
Source: BMC Plant Biol. 2019 Jun 7;19:244. doi: 10.1186/s12870-019-1857-0 (PMC6555926; doi:10.1186/s12870-019-1857-0)

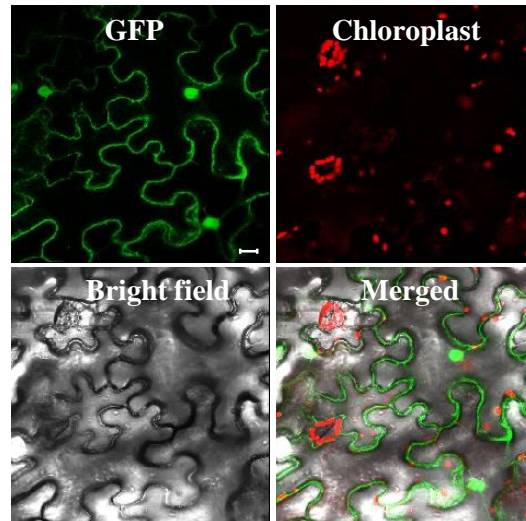

**Additional file 1**

Supplement: Supplementary file 1 — Cellular localization of the GFP only protein. GFP signals from the GFP-expressing transgenic Arabidopsis plants were observed using a confocal microscope. Red signals indicate chloroplast auto-fluorescence. Bar = 10 μm. (PDF 52 kb) [file 12870_2019_1857_MOESM1_ESM.pdf]

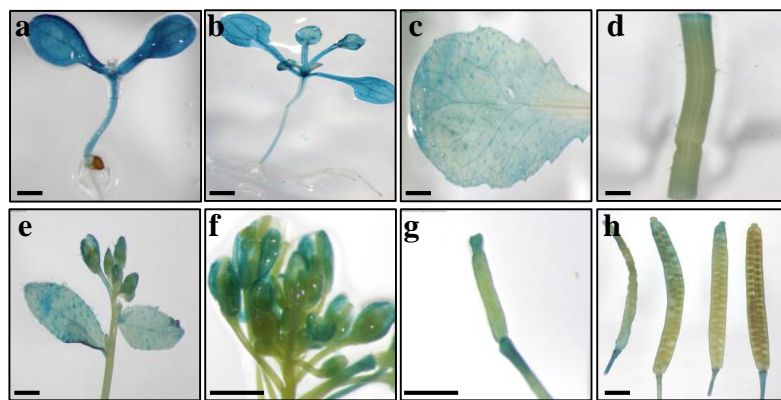

**Additional file 2**

Supplement: Supplementary file 2 — Tissue-specific expression patterns of PPR287. GUS activity in (a) 5-day-old seedling, (b) 14-day-old seedling, (c) 30-day-old leaf, (d) 40-day-old stem, (e) and (f) flowers, (g) stigma, and (h) siliques. Bar = 1 cm. (PDF 49 kb) [file 12870_2019_1857_MOESM2_ESM.pdf]

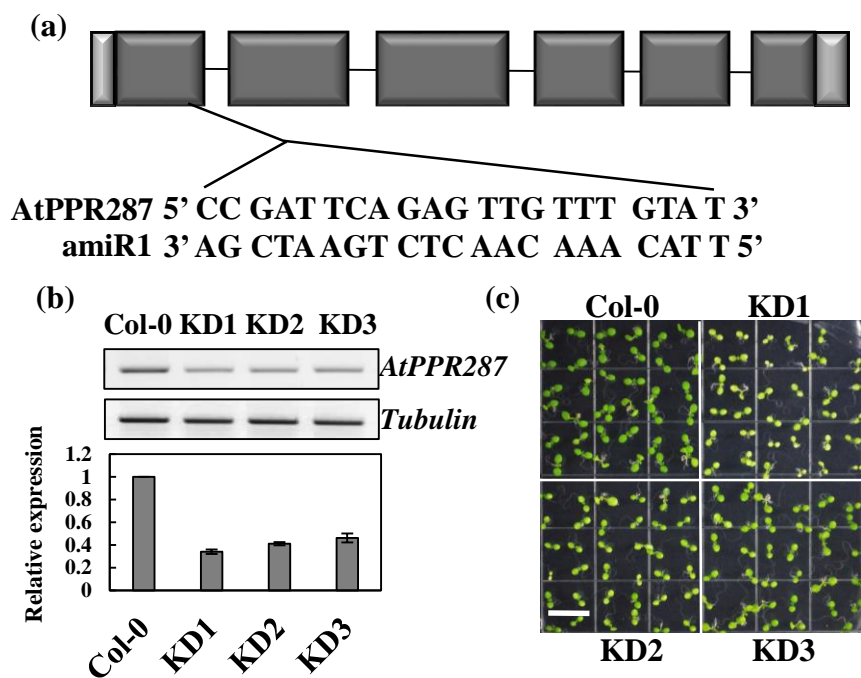

Additional file 3

Supplement: Supplementary file 3 — Development-defect phenotypes of artificial microRNA-mediated PPR287 knockdown mutants. (a) Schematic representation of the amiRNA and its target sequence. (b) Downregulation of PPR287 in each knockdown mutant line was determined by RT-PCR and real-time PCR. (c) Phenotypes of knockdown mutants on MS media at 7 days. Bar = 1 cm. (PDF 120 kb) [file 12870_2019_1857_MOESM3_ESM.pdf]

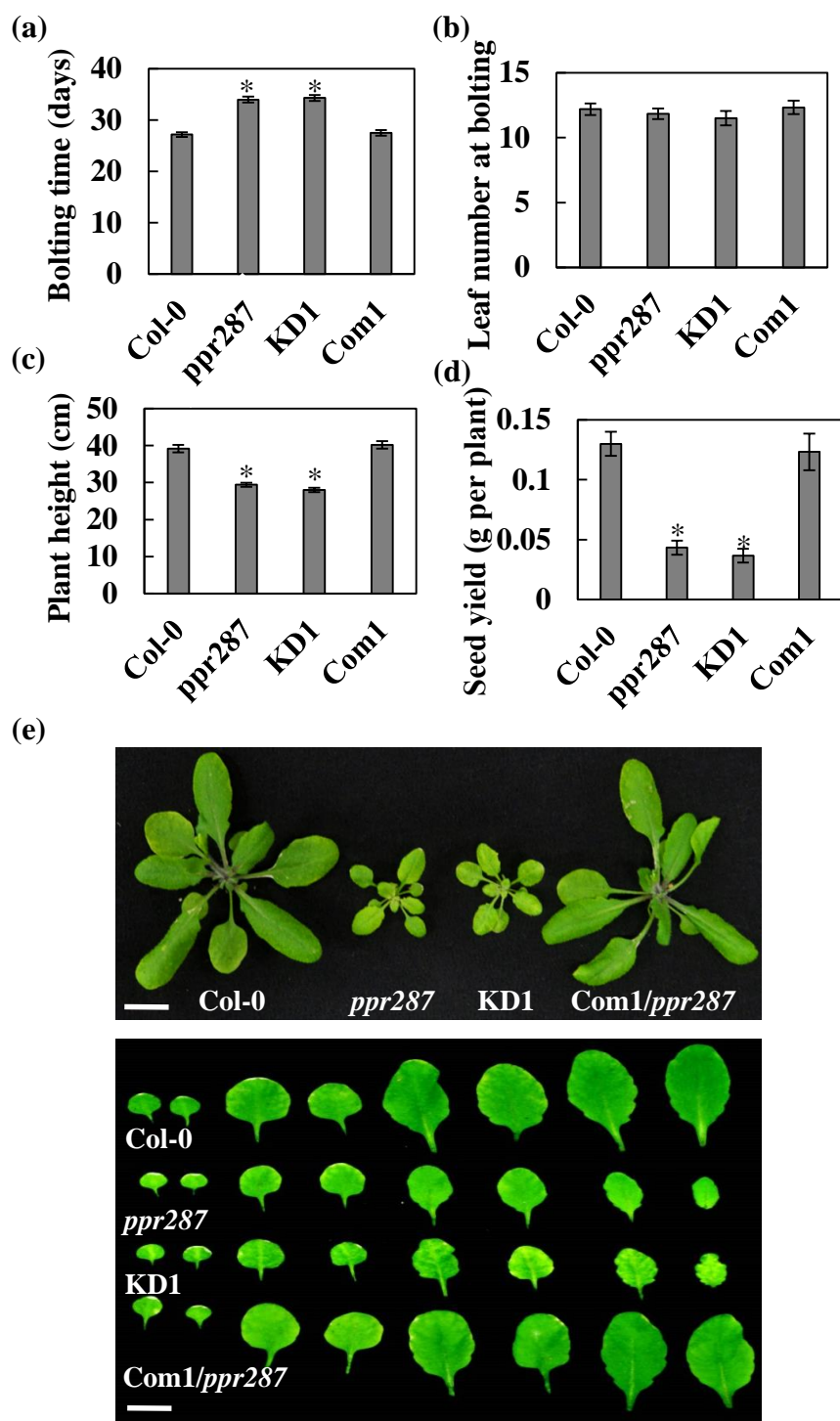

Supplement: Supplementary file 4 — PPR287 plays a role in plant growth and development. (a) Bolting time, (b) leaf number at bolting, (c) plant height, and (d) seed yield of the Col-0, ppr287 mutant, knockdown mutant (KD1), and complementation line (Com1). Values are mean ± SE of three independent experiments (n = 5), and statistically different values are indicated by asterisks (P ≤ 0.05). (e) Growth and leaf morphology at 27 days. Bar = 1 cm. (PDF 161 kb) [file 12870_2019_1857_MOESM4_ESM.pdf]

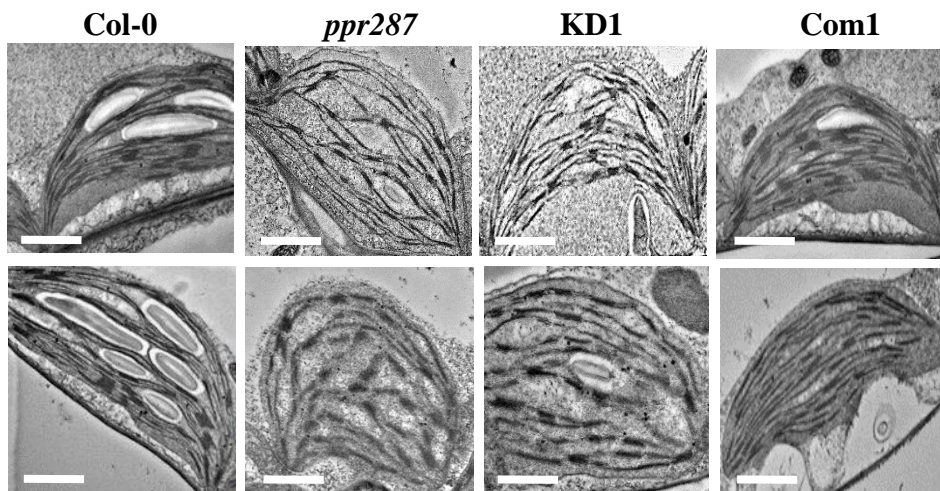

**Additional file 5**

Supplement: Supplementary file 5 — Abnormal chloroplast structures in ppr287 mutants. The Col-0, ppr287 mutant, knockdown mutant (KD1), and complementation line (Com1) were grown on MS medium, and chloroplast structures in the leaves of 3-week-old plants were observed using a transmission electron microscope. Bar = 1 μm. (PDF 177 kb) [file 12870_2019_1857_MOESM5_ESM.pdf]

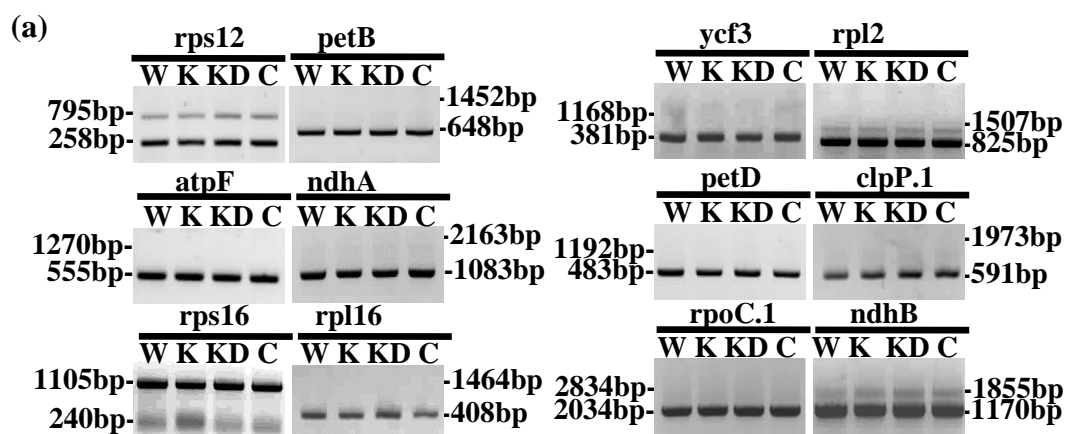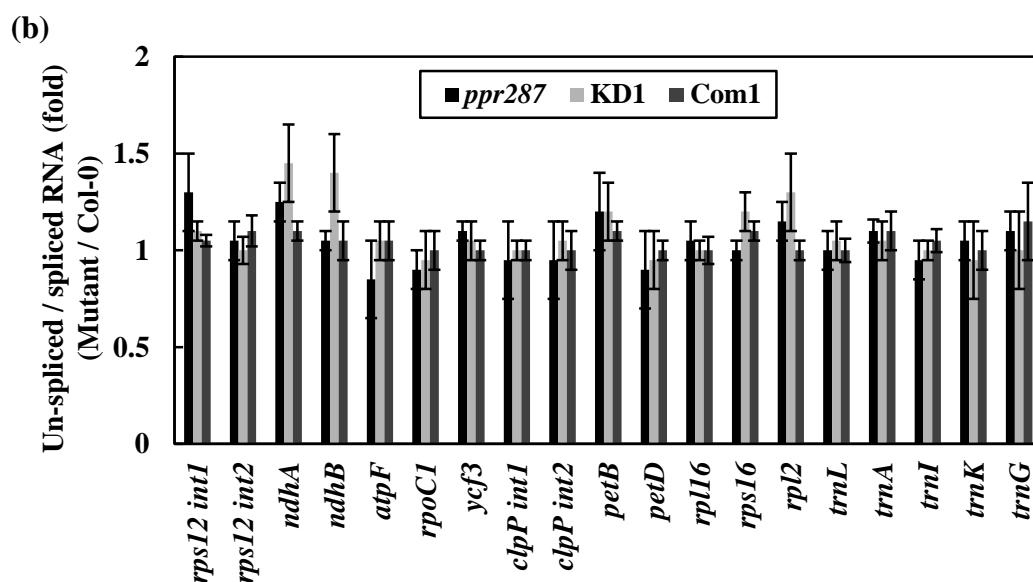

Additional file 6

Supplement: Supplementary file 6 — Splicing patterns of intron-containing chloroplast genes. (a) Total RNA was extracted from 2-week-old Col-0 (W), ppr287 mutant (K), knockdown mutant (KD1), and complementation line (Com1), and transcript levels of each gene were analyzed by RT-PCR. (b) The rations of un-spliced (precursor) to spliced (mature) transcripts between mutant and wild-type were determined by real-time RT-PCR. Data are mean ± SE of three independent biological replicates. (PDF 81 kb) [file 12870_2019_1857_MOESM6_ESM.pdf]

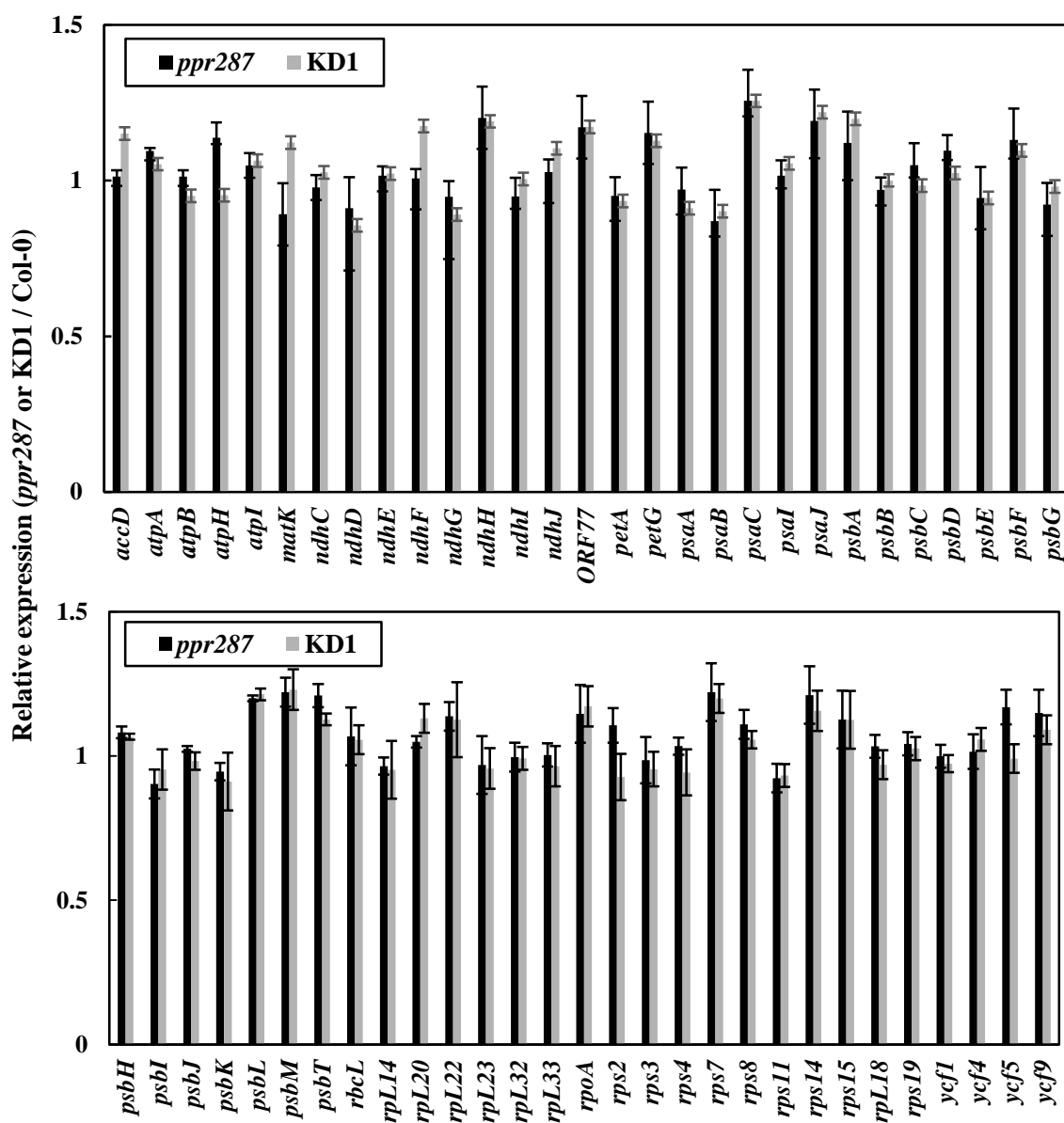

Additional file 7

Supplement: Supplementary file 7 — Expression levels of chloroplast genes. Total RNA was extracted from 2-week-old Col-0, ppr287 mutant, and knockdown mutant (KD1), and transcript levels of chloroplast genes were analyzed by real-time RT-PCR. Data are mean ± SE of three independent biological replicates (PDF 28 kb) [file 12870_2019_1857_MOESM7_ESM.pdf]
