# Supplementary material for: A chloroplast-targeted pentatricopeptide repeat protein PPR287 is crucial for chloroplast function and Arabidopsis development
Source: BMC Plant Biol. 2019 Jun 7;19:244. doi: 10.1186/s12870-019-1857-0 (PMC6555926; doi:10.1186/s12870-019-1857-0)
Supplement: Supplementary file 8 — Gene-specific primer used in RT-PCR experiments and vector construction (PDF 114 kb) [file 12870_2019_1857_MOESM8_ESM.pdf]

Additional file 8. Gene-specific primer used in RT-PCR experiments and vector construction

Gene	Primers (5′ to 3′)
PPR287-GFP vector	Forward; TCTAGAATGTTCTTTTCGTTTAGGCTATTA
	Reverse; GAATTCTAACCCCACAAGTTCTTCCGTC
PPR287-GUS vector	Forward; GCATGCGAAAGGAGACACGTTTGTTGC
	Reverse; GGATCCAACAAACGGCTTGAAAATTAC
PPR287-spanning T-DNA	Forward : ATGTCACAGGCTGTAATTTTCAAG
	Reverse : TCATAACCCCACAAGTTCTTC
PPR287-upstream T-DNA	Forward : GAACCGAATATAGTCACCTACGG
	Reverse : CTTTCTGATCAGGTGGTACCCCA
rps12	Forward : ACTATCACCCCCAAAAAACCAA
	Reverse : TTATTTTGGCTTTTTGACCCC
petB	Forward : ATGAGTAAAGTTTATGATTGGTTC
	Reverse : GGACCAGAAATACCTTGCTTAC
rpl2	Forward : ATGGCGATACATTTATACAAAAC
	Reverse : CTATTTACTACGGCGACGAAG
atpF	Forward : ATGAAAAATTTAACCGATTCTTTC
	Reverse : ATCAGTTATTTCTTTCATCGTACC
ndhA	Forward : ATGATAATTTATGCAACAGCAGTC
	Reverse : AGTGAAAAGAGTTGGAAAGAAGTG
petD	Forward :ATGGGAGTAACAAAAAAACCAG
	Reverse : CCTAAAGTTAGAGATTTATCAATCG
rpl16	Forward : ATGCTTAGTCCAAAAAGAACCAG
	Reverse : AAATAATGAATTGGGTTTTTATAGG
rpoC1	Forward : ATGATCGATCGGTATAAACATCAAC
	Reverse : TTAGGTATCATATGAACAGGCTTG
ndhB	Forward : CAGCTACTCTAGGAGGAATGTTTTTA
	Reverse : CTAGAAGCTAAAAAGGGTATCCTG
ycf3	Forward : ATGTCGGCTCAATCTGAAGG
	Reverse : TTATTCGAAGCGCCTCGTG
rps16	Forward : ATGGTAAAACTTCGTTTAAAACG
	Reverse : TCATTCCGTAAAAATCCCAGC
clpP1	Forward : CTATTGGCGTTCCAAAAGTACC
	Reverse : ACCGCTACAAGATCAACAATTCC
TUB2	Forward : CTCAAGAGGTTCTCAGCAGTA
	Reverse : TCACCTTCTTCATCCGCAGTT
