# Supplementary material for: A chloroplast-targeted pentatricopeptide repeat protein PPR287 is crucial for chloroplast function and Arabidopsis development
Source: BMC Plant Biol. 2019 Jun 7;19:244. doi: 10.1186/s12870-019-1857-0 (PMC6555926; doi:10.1186/s12870-019-1857-0)
Supplement: Supplementary file 9 — Gene-specific primers used for the analysis of splicing efficiency by real-time RT-PCR. (PDF 143 kb) [file 12870_2019_1857_MOESM9_ESM.pdf]

Additional file 9. Gene-specific primers used for the analysis of splicing efficiency by real-time RT-PCR

Gene	Primers (5′ to 3′)
rpoC1 ex1	Forward : ATGGTATTTGAAACGTCTTCCTAG
rpoC1 ex2	Reverse : ATTCAAATGAACCTCGTAATCGTAA
rpoC1 int1	Forward : AACTACTTGAGCCGGATGAGA
ycf3 ex1	Forward : TTCGGGCATTAGAACGAAAC
ycf3 ex2	Reverse : TCCAATACTCAGCGGCTTG
ycf3 int1	Forward : AGTTGGTTGTCGAGCCGTAT
clpP1 ex1	Forward : TCGAAGTCCTGGAGAAGGAG
clpP1 ex2	Reverse : AATAAGTTGATTCGAGATTTCGGT
clpP1 int1	Forward : GAACCGTATGCACCAAAAGG
clpP1 ex2	Forward : GTCGGAGGAGCAATTACCAA
clpP1 ex3	Reverse : GTGATGGTTTCGCGAAGTTT
clpP1 int2	Forward : TCATTCTGCGAAATAGAAAAACC
petB ex1	Forward : CATTGTATATTTCCGGAATATGAG
petB ex2	Reverse : TATGTTGACATGCGGAGGAA
petB int1	Forward : TCTTGGAGGGGGAGTAACCT
petD ex1	Forward : GAAGAGATAATGGATTATGGGAG
petD ex2	Reverse : GGGTTCCCCGTAATAATTGTG
petD int1	Forward : AAAAATTATCATGTCCGGTTCC
rpl16 ex1	Forward : CTTTGATATAATTGCTATGCTTAG
rpl16 ex2	Reverse : CCAAATTTTTCCACCACGTC
rpl16 int1	Forward : AAACTCTCACGTTCAGTTCTG
rpl2 ex1	Forward : AAATGGGAAATGCCCTACCT
rpl2 ex2	Reverse : GGACCTCTCCAGAAGGTAAT
rpl2 int1	Forward : GCCGTATGCTTTGGAAGAAG
rps16 ex1	Forward : AGCTGTTCTTGTTGGTTGAGC
rps16 ex2	Reverse : TTTTCTCGAGCCGTACGAGG
rps16 int1	Forward : TTCTCGAGCCGTACGAGGCCA
rps12 ex1	Forward : ATCCGAAACGTCACGAAATC
rps12 ex2	Reverse : TCTCACACCGGGTAAATCCT
rps12 int1	Reverse : GGAGCCGTATGAGGTGAAAA
rps12 ex2	Forward : CGTAAAGTTGCCAGAGTACGA
rps12 int2	Forward : TGTGGAAAGCCGTATTCGAT
rps12 ex3	Reverse : TTTGGCTTTTTGACCCCATA
atpF ex1	Forward :TACTTGGGTCACTGGCCATC
atpF int1	Forward :TTCGGGAAGGGATCATAGAA
atpF ex2	Reverse : GCTCCTTCACGCAGTTCTTC
ndhA ex1	Forward :TTAGGTGGTCTGCGAGCTG
ndhA int1	Forward :AGGCCAAGACCTCATGTACG
ndhA ex2	Reverse : TTGACGCCACAAATTCCAT
ndhB ex1	Forward :TCATCAATGGACTCCTGACG
ndhB int1	Forward :AGTCTCATGCACGGTTTTGA
ndhB ex2	Reverse : CCAGAAGAAGATGCCATTCA
trnL ex1	Forward : GGGGATATGGCGGAATTGG
trnL int1	Forward : GGAACTTAATTAATCGGACGAG
trnL ex2	Reverse : TGGGGATAGAGGGACTTGAAC
trnA ex1	Forward : GGGGATATAGCTCAGTTGGTAG
trnA int1	Forward : AGAACCACAAGAATCCTTAG

trnA ex2	Reverse : TGGAGATAAGCGGACTCGAAC
trnI ex1	Forward : GGGCTATTAGCTCAGTGGTAGA
trnI int1	Forward : GGGAACAAGCACACTTGGAG
trnI ex2	Reverse : TGGGCCATCCTGGATTTGA
trnK ex1	Forward : GGGTTGCTAACTCAACGGTAG
trnK int1	Forward : ATAGAGAAAGCTGTGTGCAATGA
trnK ex2	Reverse : TGGGTTGCCCGGGACTCG
trnG ex1	Forward : GCGGGTATAGTTTAGTGGTAAAA
trnG int1	Forward : AGCTCACATAGATGTTATGG
trnG ex2	Reverse : AGCGGGTAGCGGGAATCG
