# Supplementary material for: A chloroplast-targeted pentatricopeptide repeat protein PPR287 is crucial for chloroplast function and Arabidopsis development
Source: BMC Plant Biol. 2019 Jun 7;19:244. doi: 10.1186/s12870-019-1857-0 (PMC6555926; doi:10.1186/s12870-019-1857-0)
Supplement: Supplementary file 10 — Gene-specific primers used for the analysis of expression levels of chloroplast genes by quantitative RT-PCR. (PDF 234 kb) [file 12870_2019_1857_MOESM10_ESM.pdf]

Additional file 10. Gene-specific primers used for the analysis of expression levels of chloroplast genes by quantitative RT-PCR

Gene	Primers (5′ to 3′)
accD	Forward : GGTTCAATTTTATGTTTTCTAAGG
	Reverse : GCATGGAGCTGAAATAACTCG
atpA	Forward : CCATTAGAGCCGACGAAATTAG
	Reverse : AGTAGGAAACGTTCTAGTTGCTC
atpB	Forward : ATCCTACTACTTCAAATCCAGAGG
	Reverse : ATTTCTTCAATTTACTCTCCATTTC
atpH	Forward : ATGAATCCACTGGTTTCTGCTG
	Reverse : AAAAGGATTCGCAAATAAAAGC
atpI	Forward : AATGTTTTATCATGTTCCATCAAC
	Reverse : TTAATGATGACCTTCCATAGACTC
matK	Forward : AAGAAAATAAAGACTTTACCTTCAG
	Reverse : ATCCAAATACCAAATTCGCACTC
ndhC	Forward : ATGTTTCTGCTTTACGAATATGA
	Reverse : CTAAGACCATTCCAATGCTCC
ndhD	Forward : GCTGGTTCATTAATGTTATTTCTC
	Reverse : GGATTCGACCTTGTCACTTGC
ndhE	Forward : ATACTCGAACATGTACTTGTTTTGAG
	Reverse : TTACTTATTTAATAAGGTCGATTGG
ndhF	Forward : ATATTCATGGATCATCCCTTTC
	Reverse : AAGAACAGATAAGAAGAGATGCG
ndhG	Forward : ATGGATTTGCCTGGACCAATAC
	Reverse : TATTGACGAGCCACAGAAATTG
ndhH	Forward : GAGACCAGTTACAGGAAAAGATC
	Reverse : TTAACGATCAACTTCTCCCATA
ndhI	Forward : ATGCTTCCTATGATAACCGGG
	Reverse : TCAAATTAAAGGGTTTACCCC
ndhJ	Forward : CACTTTGTCCGTTTGGCTAG
	Reverse : CATCTTGTATTTCATAAAAATTGG
ORF77	Forward : ATGCTACTACTGAAACATGGAAGA
	Reverse : GTTATTCAGTTGATTCGTTATTGG
petA	Forward : ATGCAAACTAGAAATACCTTTTC
	Reverse : CTAAAAATTCATTTCGGATAATTG
petG	Forward : ATGATTGAAGTTTTTTTATTTGG
	Reverse : TTAAAAGTCCAACTGATCACCAC
psaA	Forward : GATTATTCGTTCGCCGGAAC
	Reverse : AGAAGAACGCCCATGTTGTG
psaB	Forward : ATGGCATTAAGATTTCCAAGG
	Reverse : TTAACCGAATTTGCCCGATG
psaC	Forward : ATGTCACATTCAGTAAAAATTTATG
	Reverse : TCAATAAGCTAGACCCATACTTC
psaI	Forward : ATGACAACTTTCAATAACTTACCC
	Reverse : TCTTATTTTTTTGAATATGAAGAAA
psaJ	Forward : ATGCGAGATCTAAAAACATATC
	Reverse : TAGAATGAAAAAAAGGGAAATG
psbA	Forward : ATGACTGCAATTTTAGAGAGACG
	Reverse : TAGATGGAGCCTCAACAGCAG
psbB	Forward : ATGGGTTTGCCTTGGTATCG

	Reverse : TCAGACTGCTTGTCGTTTTGTAG
psbC	Forward : ATGAAAACCTTATATTCCCTGAGG
	Reverse : AGTTAAGAGGAGTCATGGAAAGAAC
psbD	Forward : ACTATAGCCCTTGGTAAATTTACC
	Reverse : AAAGAGCGTTTCCACGTGGTAG
psbE	Forward : ATGTCTGGAAGCACAGGAGAAC
	Reverse : CTAAATTCATCGAGTTGTTCCAAAG
psbF	Forward : ATGACTATAGATAGGACCTATCCAA
	Reverse : TATCGTTGGATGAACTGCATTG
psbG	Forward : GAATTCCATTAAGTTTCCCATAC
	Reverse : CTAATTCACTAATTCGTGGGACG
psbH	Forward : ATGGCTACACAAACTGTTGAAG
	Reverse : CTAATTCACTGAAATTCCATCC
psbI	Forward : ATGCTTACTCTCAAACTTTTTG
	Reverse : ATTCTTCACGTCCCGGATTAC
psbJ	Forward : ATGGCTGATACTACTGGAAGGA
	Reverse : CTACAGGGATGAACCTAATCCTG
psbK	Forward : ATGCTTAATATATTTAATTTGATCTG
	Reverse : TTATCGAAAACTTACAGCGGC
psbL	Forward : ATGACACAATCAAATCCGAACG
	Reverse : TTAATTGAAGAAATAATTCGAAAATA
psbM	Forward : ATGGAAGTAAATATTCTTGCA
	Reverse : TTAATCATTTTGACTAACGG
psbT	Forward : ATGGAAGCATTGGTTTATACATTTC
	Reverse : TCATTTTTTAGTTGAAATTTTAGG
rbcL	Forward : TCACCACAAACAGAGACTAAAGC
	Reverse : ACTCTTGGCCATCTAATTTATCG
rpl4	Forward : AAACCTATTTGAATGTAGCAGAC
	Reverse : TTATAATACCTCAGGAGCTAATGA
rpl18	Forward : GAATAAATCTAAGCGACTTTTTAC
	Reverse : TATTTTTTTCTGGTTCTAAGACTAG
rpl20	Forward : ATGACTAGAATTAAACGCGGA
	Reverse : TTATGTCATTCGAAATTGTATAAAG
rpl22	Forward : AGAGAAAAAAGAAATCATATACGG
	Reverse : TATTTTTTGTCCCATAGGCCTC
rpl23	Forward : ATGGATGGAATCAAATATGCAG
	Reverse : GTTCTTTTCTTTCTAAGAGGTGG
rpl32	Forward : GGCAGTTCCAAAAAAACGTAC
	Reverse : AAAAACTTTTTGAATTACCTGTAG
rpl33	Forward : ATGGCCAAGGGTAAAGATGTTC
	Reverse : CTATTTCTTGATTTCCCCGTG
rpoA	Forward : ATGGTTCGAGAGAAAGTCAAAG
	Reverse : CTAATATCTTTTTTACATCTTCTACG
rps2	Forward : ATGACAAAAAGATATTGGAACATCG
	Reverse : GTATATAGCTAGAACGGCCCTCAC
rps3	Forward : ATGGGACAAAAAATAAATCCAC
	Reverse : CGTCTACGAATATCCAAATTTTTA
rps4	Forward : ATGTCACGTTACCGAGGGCC
	Reverse : TCGAGAGTAATATTCTACGACAAGC
rps7	Forward : ATGTCACGCCGAGGTACTGC
	Reverse : TTAACGAAAATGTGCAAAAGCTC
rps8	Forward : GGGGAAAGACACCATTGCTG
	Reverse : CCATATATAACACAAAATTTCTCCG
rps11	Forward : ATGGCAAAACCTATATTAAGAATTG

	Reverse : CTATACACGTCTTTTTTTAGGGG
rps14	Forward : ATGGCAAAGAAAAGTTTGATTTA
	Reverse : TTACCAGCTTGATCTTGTTGC
rps15	Forward : GATAAAAAATATAGTCATTTCATTTG
	Reverse : GTTTTTAATTCCCGAATATTCAAC
rps19	Forward : GTGACACGTTCACTAAAAAAAAAC
	Reverse : CGACGAGATCTATTATCATTTTTG
ycf1	Forward : ATGGTTTTTCAATCTTTTATACTAGG
	Reverse : CTAAATTCGAATTTTCTTTATTTCC
ycf4	Forward : ATGAGTTGGCGATCAGAATCTA
	Reverse : TCAAAATACTTCAATTGGTACACG
ycf5	Forward : ATGATTTTTTCAATTTTAGAGCA
	Reverse : AATTCGATGTAAATGAACCATAAC
ycf9	Forward : ATGACTATTGCTTTCCAATTGG
	Reverse : AGAGATAAGAGAATTAAGGATACCC
